# A Population-Based Comparison of the AJCC 7th and AJCC 8th Editions for Patients Diagnosed with Stage III Cutaneous Malignant Melanoma in Sweden

**DOI:** 10.1245/s10434-019-07448-y

**Published:** 2019-05-20

**Authors:** Karolin Isaksson, Dimitrios Katsarelias, Rasmus Mikiver, Ana Carneiro, Lars Ny, Roger Olofsson Bagge

**Affiliations:** 1 Department of Clinical Sciences Lund, Surgery, Lund University, Skåne University Hospital, Lund, Sweden; 2 Department of Surgery, Institute of Clinical Sciences, Sahlgrenska Academy at the University of Gothenburg, Sahlgrenska University Hospital, Gothenburg, Sweden; 3 Department of Clinical and Experimental Medicine, Regional Cancer Center South East Sweden, Linköping University, Linköping, Sweden; 4 Department of Clinical Sciences Lund, Oncology, Lund University, Skåne University Hospital, Lund, Sweden; 5 Department of Oncology, Institute of Clinical Sciences, Sahlgrenska Academy at the University of Gothenburg, Sahlgrenska University Hospital, Gothenburg, Sweden; 6 Wallenberg Centre for Molecular and Translational Medicine, University of Gothenburg, Gothenburg, Sweden

## Abstract

**Background:**

Cutaneous melanoma is steadily increasing worldwide. The new AJCC 8th edition was recently launched and introduced several changes in melanoma staging, particularly for stage III. We conducted a population-based registry study with the purpose to evaluate the impact and prognostic accuracy of the new classification in Sweden.

**Methods:**

Consecutive patients diagnosed with stage III melanoma between January 2005 and September 2017 were identified by the Swedish Melanoma Registry (SMR) and included for analyses. Patients with multiple primary melanomas were excluded. Patients were classified according to the AJCC 7th as well as the 8th edition. Melanoma-specific survival (MSS) was retrieved from the Swedish Cause of Death Registry.

**Results:**

A total of 2067 eligible patients were identified from the SMR; 1150 patients (57%) changed stage III subgroup when reclassified according to the AJCC 8th edition. The median 5- and 10-year MSS for the whole cohort of stage III melanoma patients was 59% and 51% respectively. The MSS for substage IIIA, B, and C were all improved when patients were reclassified by using to the AJCC 8th edition. The newly defined substage IIID had the worst prognosis with a 10-year MSS of 16%.

**Conclusions:**

A high proportion of patients diagnosed with stage III melanoma in Sweden between 2005 and 2017 was restaged to another subgroup, when they were reclassified according to the AJCC 8th of staging manual. We established an improved MSS for all substages compared with the former AJCC 7th edition. This may have implications on decisions about adjuvant treatment.

The incidence of cutaneous malignant melanoma is steadily increasing in most countries with fair-skin populations. In several countries, the increase has been reported as up to 5% annually.^1^^,^^2^ In Sweden, melanoma incidence has reached 40 cases per 100,000 people per year and is the fifth most common cancer with approximately 4000 new invasive melanomas diagnosed in 2017.^2^ The majority of patients are diagnosed with thin melanomas (≤ 1 mm), generally having a very favourable prognosis.^3^–^9^ With increasing Breslow thickness, the risk of being diagnosed with stage III disease increases. According to the American Joint Committee on Cancer (AJCC) staging manual, patients with satellite metastasis (including microsatellites within the primary melanoma), in-transit metastasis, and/or regional lymph node disease are classified as stage III melanoma. The majority of patients diagnosed with regional lymph node metastasis have occult disease, i.e., a positive sentinel lymph node (SLN). The overall risk of a positive sentinel lymph node is approximately 20% and the risk of node positivity increases with increasing Breslow thickness.^10^–^12^ AJCC melanoma staging system further classifies patients with stage III disease into different subgroups according to prognosis. Recently the AJCC 7th edition was replaced with the 8th edition, and the number of subgroups were revised from three (A–C) to four (A–D) groups.^6^^,^^13^ Indeed, in the revised 8th edition stage III classification was modified implementing changes in T and N classification, which impact staging, leading to possible substage migration. The changes for the T status include: a redefinition of T1a and T1b melanoma and also a reduction in decimals from two to one in reporting Breslow thickness defining the T status, and T1 subclassification no longer depends on mitotic rate. Regarding the N status non-nodal regional disease, including microsatellites, satellites, and in-transit cutaneous metastases, is more formally stratified by N category according to the number of tumor-involved lymph nodes.^8^

During the last years, new, effective systemic treatments have been introduced and are available and approved for advanced unresectable stage III disease and for stage IV patients. In the past decade, three checkpoint inhibitors (ICIs) have been established for use in advanced melanoma, i.e., the CTLA-4 inhibitor ipilimumab and the PD-1 inhibitors nivolumab and pembrolizumab, respectively. In addition, there are targeted therapies (BRAF/MEK inhibitors) available as a treatment for patients with BRAF-mutated melanomas. Taken together, these medical therapies have remarkably changed the prognosis for these patient groups. Recently, several trials have shown a relapse free survival benefit of systemic treatment in stage III melanoma, leading to a change in the therapeutic approach of radically operated stage III patients, with approval and implementation of adjuvant treatment in many countries including Sweden.^14^–^18^ However, all of these adjuvant trials were performed using the former AJCC 7th edition for stage classification. According to the most recent AJCC 8th edition, the 5-year melanoma-specific survival (MSS) for stage IIIA-C is significantly improved compared with the 5-year MSS in the 7th edition. This study was designed to perform a population-based validation of stage III classification according to the AJCC 8th edition classification by assessing survival differentiation observed in the 7th and 8th editions and melanoma-specific survival.

## Patients and Methods

Patients diagnosed with stage III cutaneous malignant melanoma classified according to either the AJCC 7th or AJCC 8th edition, between January 1, 2005 and September 30, 2017, were retrospectively retrieved from the population-based and prospectively collected database Swedish Melanoma Registry (SMR). All changes performed in the AJCC 8th edition concerning stage III definitions were respected when reclassifying the individual patients. For exact substage classification according to the different editions, we kindly refer to AJCC 7th and AJCC 8th edition of Melanoma Staging Manuals, respectively. Patients with multiple melanomas were excluded from the study. Age, sex, localisation of primary tumor, histologic subtype, Breslow thickness, ulceration, and mitoses were collected for all patients. Patients were followed up until December 31, 2017, and the melanoma-specific survival (MSS) was calculated using the Swedish Cause of Death Registry.

### Statistical Analysis

Survival and 95% confidence intervals were estimated using the Kaplan–Meier method. The log-rank test was performed to compare survival curves according to the AJCC 7th and AJCC 8th editions. A two-tailed *p* value of < 0.05 was considered statistically significant. All analyses were performed using IBM SPSS statistic version 22.0 (IBM Corp., Armonk, NY) and R version 3.3.1 (The R Foundation for Statistical Computing).

## Results

**Fig. 1:**
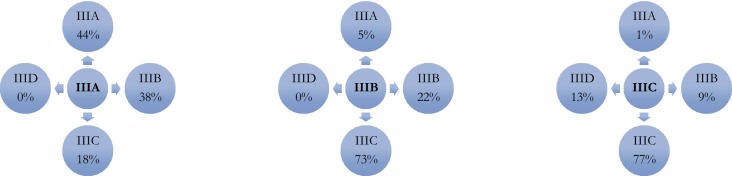
Illustration of substage migration from AJCC 7th edition (in central circles) to AJCC 8th edition (peripheral circles)

**Fig. 2:**
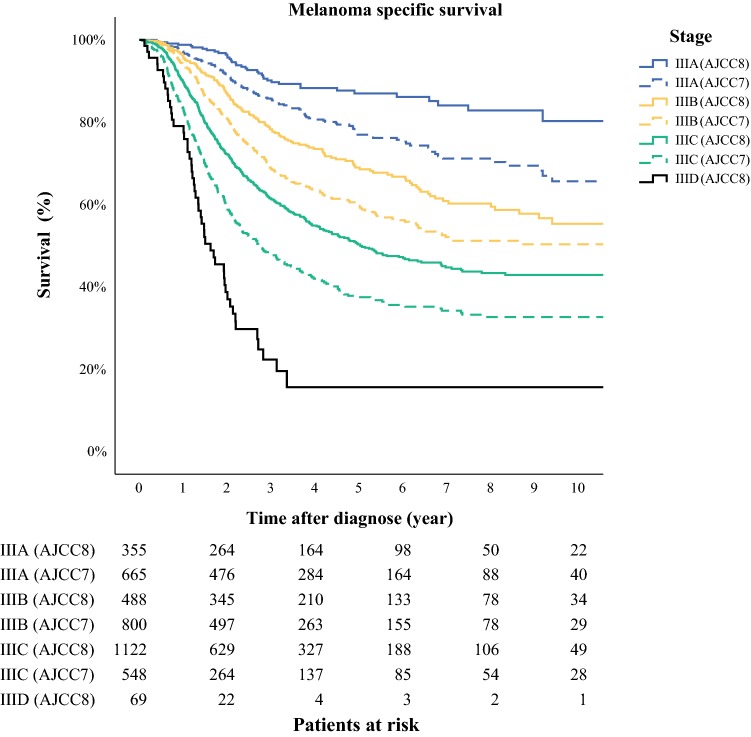
Melanoma-specific survival compared between AJCC 7th and AJCC 8th editions

A total of 2067 patients with cutaneous melanoma were eligible and included in the study. Median age at diagnosis was 65 years, and the majority were male (59%). The median Breslow thickness was 3.1 mm, and ulceration was present in 51% of the primary melanomas. The trunk and extremities were the most frequent localisations of the primary melanoma, and superficial spreading melanoma and nodular melanoma were the dominating histologic subtypes (Table 1). Stage IIIB was the most common substage (40%) when the AJCC 7th edition was used and IIIC the most common substage (55%) when the patients were classified according to the AJCC 8th edition. A total of 1150 patients (57%) changed stage III subgroup when reclassified from the AJCC 7th to the AJCC 8th edition. Thirty-eight percent of patients classified as stage IIIA according to the AJCC 7th edition were reclassified as stage IIIB according to the AJCC 8th edition, whereas 18% were upstaged to IIIC when reclassified. Seventy-nine percent of the patients classified as IIIB according to the AJCC 7th edition were reclassified to another substage when the AJCC 8th edition was used, the majority (73%) being upstaged to stage IIIC. Finally, in IIIC according to the AJCC 7th edition 28% of the patients changed substage whereof 13% were classified as the new IIID substage according to the AJCC 8th edition (Table 2; Fig. 1). The median 5- and 10-year MSS for the whole cohort with stage III melanoma, regardless of classification edition used, was 59% and 51% respectively. The survival rates for substage IIIA, B, and C were all improved when patients were reclassified according to the AJCC 8th edition. When analysing the difference between the two staging systems, stage IIIA in the AJCC 8th edition had a significantly better MSS with a 5-year and 10-year MSS of 87% and 80% respectively compared with 77% and 66% respectively according to the AJCC 7th edition. The 5- and 10-year MSS for subgroup IIIB improved from 60% and 50% respectively to 69% and 55% respectively when staging according to the AJCC 7th edition and AJCC 8th edition were compared. For patients in the IIIC substage, the 5-year MSS was 38% and 10-year MSS was 33% when the AJCC 7th edition classification was used and 50% and 43% respectively when classified according to the AJCC 8th edition. The improvement in survival according to the 8th edition was statistically significant for substages IIIA, B, as well as C (*p* < 0.001, log-rank; Table 3; Fig. 2). The new defined substage IIID, according to the AJCC 8th edition, had the worst prognosis with a 10-year MSS of 16% (Fig. 2).

**Table 1:** Patient and primary melanoma characteristics

Parameter	Number (%)	Median (IQR)
Total	2067	
Sex		
Female	849 (41)	
Male	1218 (59)	
Age		65 (51–74)
Breslow (mm)		3.1 (1.9–5.0)
≤ 1	120 (6)	
1–2	483 (23)	
2–4	693 (34)	
> 4	761 (37)	
Unknown	10 (0)	
Ulceration		
Yes	1062 (51)	
No	972 (47)	
Missing	33 (2)	
Mitoses		
Yes	1254 (61)	
No	112 (5)	
Missing	701 (34)	
Localisation (primary)		
Head and neck	172 (8)	
Extremity	946 (46)	
Trunk	941 (46)	
Missing/unknown	8 (0)	
Histologic subtype		
SSM	856 (41)	
NM	838 (41)	
LMM	32 (2)	
ALM	66 (3)	
Missing/unknown	243 (12)	
N status		
SLNB performed	1606 (78)	
SLN+ (occult)	1519 (73)	
Clinical lymph nodes	546 (26)	
Matted lymph nodes	30 (1.5)	
Intransit/satellites	204 (10)	

*SSM* superficial spreading melanoma, *NM* nodular melanoma, *LMM* lentigo maligna melanoma, *ALM* acral lentiginous melanoma, *SLNB* sentinel lymph node biopsy, *SLN* sentinel lymph node

**Table 2:** Crosstabulation comparing classification of patients according to AJCC 7th and AJCC 8th editions

	AJCC 8th edition						
		III-	IIIA	IIIB	IIIC	IIID	Total
	III-	0	12	19	23	0	54
	IIIA	0	295	253	117	0	665 (33%)
AJCC 7th edition	IIIB	0	42	172	586	0	800 (40%)
	IIIC	33	6	44	396	69	548 (27%)
	Total	33	355 (17%)	488 (24%)	1122 (55%)	69 (3%)	2067

III- consists of not classifiable stage III and are not included in the calculations of percentage for the different substages

**Table 3:** Melanoma specific survival according to the AJCC 7th and AJCC 8th editions

Year	Stage	Stage classification	
		AJCC 7	AJCC 8
		MSS, % (95% CI)	MSS, % (95% CI)
5 year MSS	IIIA	77 (73.1–81.1)	87 (82.8–91.4)
	IIIB	60 (55.6–64.0)	69 (64.3–74.0)
	IIIC	38 (33.4–42.8)	50 (46.9–54.0)
10 year MSS	IIIA	66 (59.4–72.5)	80 (73.2–88.1)
	IIIB	50 (45.5–55.6)	55 (48.9–62.6)
	IIIC	33 (28.1–37.9)	43 (39.0–47.1)

*CI* confidence interval, *MSS* melanoma specific survival

## Discussion

The purpose of this study was to validate and evaluate the impact of the implementation of the new AJCC 8th edition for cutaneous melanoma stage III patients in a population-based cohort. We found that a substantial number of patients were restaged when reclassified according to the AJCC 8th edition. The highest proportion of reclassified patients was seen in stage IIIB, where the majority of patients changed substage. Moreover, the majority of stage III patients were classified as stage IIIB in the former AJCC 7th edition, a finding that was altered to IIIC according to the AJCC 8th edition and in line with the results of the analysis from International Melanoma Database and Discovery Platform (IMDDP), on which the results in the AJCC 8th edition are based. The distribution concerning the different substages within stage III as per the AJCC 8th edition also was similar in our study.

We confirmed improved 5- and 10-year MSS for the former stage III subgroups when reclassified according to the AJCC 8th edition, albeit MSS rates in the current study are lower than the ones presented in the AJCC 8th Edition Cancer Staging Manual.^8^ At the American Society of Clinical Oncology meeting in 2018, Madu et al. presented a validation of the AJCC 8th edition based on an institutional database from the Netherlands. The 5-year MSS survival rates for the different stage III subgroups from that analysis were more consistent with our results.^19^ However, there are differences between the IMDDP, the Dutch database, and the present patient cohort, especially in what concerns the time period of inclusion, the total number of patients, and the time of follow-up. More importantly, our cohort is the only retrieved from a prospective, national, population-based registry.

Patients diagnosed with invasive malignant melanoma in Sweden are registered in the SMR, using the Information Network for Cancer Care (INCA) webportal. The coverage of the Swedish population data is nearly complete (99%), and the registry has complete follow-up data on overall and melanoma-specific survival through continuous annual updates from the Swedish Cause of Death Registry.^20^ The SMR covers well information on details from the primary melanoma and results of performed sentinel lymph node biopsy and subsequent complete lymph node dissection (CLND) when performed for positive SLN. Data for clinically detected stage III disease is not fully covered in the registry as recurrent disease or primary clinical stage III disease without a known primary cutaneous melanoma is not mandatorily reported to the SMR until now. Thus, clinical detected stage III patients are potentially missed in the present analysis due to underreporting although the number of such patients would most probably be low and not significantly impact the analysis.

In Sweden, standard of care for stage III melanoma patients has besides radical surgery usually not included any other treatment for most patients. In some cases, patients with an estimated high risk for regional relapse, adjuvant radiotherapy (RT) against a positive lymph node basin after lymph node dissection has been recommended. Adjuvant RT can decrease the incidence of regional lymph node recurrence in patients with high-risk regional node involvement, but there is no evidence of any improvement in overall survival.^21^^,^^22^ The approval by the FDA of ipilimumab as adjuvant treatment after radical surgery for stage III melanoma in 2015 was based on a clinical study conducted using the AJCC 7th edition. The same applies to the more recent trials of PD1-inhibitors and BRAF/MEK inhibitors in the adjuvant setting. Recently, both nivolumab and pembrolizumab were approved for adjuvant treatment in Europe for all stage III (IIIA–D).^23^ Several countries also have approved adjuvant treatment for melanoma stage III, with no subgroup limitation. In some countries, approval of adjuvant treatment for stage IIIA has been limited to pembrolizumab, as the EORTC/Keynote 054 trial included stage IIIA (according to the AJCC 7th edition), although a tumor burden of >1 mm in the sentinel node was required.

Moreover, the adjuvant trials leading to approval required all patients with positive sentinel lymph node to have CLND performed, which no longer is the current clinical standard.^15^–^18^ Parallel with the adjuvant studies and the revision of the former AJCC 7th to AJCC 8th edition, there has been crucial changes in the surgical treatment of clinically occult node-positive stage III melanoma. Because both the MSLT II and the DeCOG trials showed no survival benefit from CLND after positive sentinel lymph node, there is an overall consensus to omit CLND in this situation and instead schedule the patient for close clinical and radiological follow-up (ultrasonography).^24^^,^^25^

An update of the EORTC/Keynote 054 trial, presented at the 2018 Society for Melanoma Research Congress, showed that only 8% of the patients would be classified as stage IIIA according to the AJCC 8th edition compared with 15% according to the AJCC 7th edition used in the trial. Moreover, this analysis showed that the 1-year, recurrence-free survival in the stage IIIA subgroup was 92.7% versus 92.5% in the placebo arm. The AJCC 8th edition was found to have a prognostic importance for all stage III subgroups but no predictive importance for the treatment comparison regarding recurrence-free survival.^26^ These differences are important when considering the effects of adjuvant therapies on survival. For example, in the adjuvant ipilimumab trial, there was an overall survival benefit at 5 years with a hazard ratio of 0.72 in favor of ipilimumab compared with placebo for all stage III patients.^14^ When applying this relative risk on the current data for the Swedish stage IIIA population, this gives an absolute survival increase at 5 years of 6.4% using the AJCC 7th edition compared with 3.6% using AJCC 8th edition, increasing the number needed to treat from approximately 17 to 25 patients. These findings highlight the importance of analysing the adjuvant trials in light of the AJCC 8th edition, especially in follow-up updates with recurrence-free and overall survival data.

## Conclusions

A high proportion of patients diagnosed with stage III cutaneous melanoma in Sweden, between 2005 and 2017, was restaged when reclassified according to the AJCC 8th edition. We established an improved melanoma-specific survival for all substages compared with classification according to the former AJCC 7th edition. As other authors, we identified difficulties concerning the introduction of a new staging classification while practice changing adjuvant trials have been conducted based on a different staging classification, along with substantial change in the surgical management of this patient group. With the introduction of adjuvant treatment for stage III melanoma patients, continuous work is needed to identify those patients who will benefit from the treatment. This will certainly need additional studies to address other prognostic factors, such as sentinel node tumor burden and tumor biomarkers, besides the current parameters today defining a stage III patient. Moreover, further validation and analysis of already existing institutional and population-based prognostic models will likely be an additional important tool in clinical decision-making and thereby allow better patient counselling and selection for adjuvant treatment in stage III melanoma.^27^–^30^
